# Associations of Insurance Churn and Catastrophic Health Expenditures With Implementation of the Affordable Care Act Among Nonelderly Patients With Cancer in the United States

**DOI:** 10.1001/jamanetworkopen.2021.24280

**Published:** 2021-09-08

**Authors:** Benjamin B. Albright, Fumiko Chino, Junzo P. Chino, Laura J. Havrilesky, Emeline M. Aviki, Haley A. Moss

**Affiliations:** 1 Department of Obstetrics and Gynecology, Duke University Medical Center, Durham, North Carolina; 2 Department of Radiation Oncology, Memorial Sloan Kettering Cancer Center, New York, New York; 3 Department of Radiation Oncology, Duke University Medical Center, Durham, North Carolina; 4 Department of Surgery, Memorial Sloan Kettering Cancer Center, New York, New York

## Abstract

**Importance:**

Health insurance coverage is dynamic in the United States, potentially changing from month to month. The Patient Protection and Affordable Care Act (ACA) aimed to stabilize markets and reduce financial burden, particularly among those with preexisting conditions.

**Objective:**

To describe the risks of insurance churn (ie, gain, loss, or change in coverage) and catastrophic health expenditures among nonelderly patients with cancer in the United States, assessing for changes associated with ACA implementation.

**Design, Setting, and Participants:**

This retrospective, cross-sectional study uses data from the Medical Expenditure Panel Survey, a representative sample of the US population from 2005 to 2018. Respondents included were younger than 65 years, identified by health care use associated with a cancer diagnosis code in the given year. Statistical analysis was conducted from July 30, 2020, to January 5, 2021.

**Exposures:**

The Patient Protection and Affordable Care Act.

**Main Outcomes and Measures:**

Survey weights were applied to generate estimates for the US population. Annual risks of insurance churn (ie, any uninsurance or insurance change or loss) and catastrophic health expenditures (spending >10% income) were calculated, comparing subgroups with the adjusted Wald test. Weighted multivariable linear regression was used to assess for changes associated with ACA implementation.

**Results:**

From 6069 respondents, we estimated a weighted mean of 4.78 million nonelderly patients (95% CI, 4.55-5.01 million; female patients: weighted mean, 63.9% [95% CI, 62.2%-65.7%]; mean age, 50.3 years [95% CI, 49.7-50.8 years]) with cancer annually in the United States. Patients with cancer experienced lower annual risks of insurance loss (5.3% [95% CI, 4.5%-6.1%] vs 7.6% [95% CI, 7.4%-7.8%]) and any uninsurance (14.6% [95% CI, 13.3%-16.0%] vs 24.1% [95% CI, 23.5%-24.7%]) but increased risk of catastrophic health expenditures (expenses alone: 12.4% [95% CI, 11.2%-13.6%] vs 6.3% [95% CI, 6.2%-6.5%]; including premiums: 26.6% [95% CI, 25.0%-28.1%] vs 16.5% [95% CI, 16.1%-16.8%]; *P* < .001) relative to the population without cancer. Patients with cancer from low-income families and with full-year private coverage were at particularly high risk of catastrophic health expenditures (including premiums: 81.7% [95% CI, 74.6%-88.9%]). After adjustment, low income was the factor most strongly associated with both insurance churn and catastrophic spending, associated with annual risk increases of 6.5% (95% CI, 4.2%-8.8%) for insurance loss, 17.3% (95% CI, 13.4%-21.2%) for any uninsurance, and 37.4% (95% CI, 33.3%-41.6%) for catastrophic expenditures excluding premiums (*P* < .001). In adjusted models relative to 2005-2009, full ACA implementation (2014-2018) was associated with a decreased annual risk of any uninsurance (−4.2%; 95% CI, −7.4% to −1.0%; *P* = .01) and catastrophic spending by expenses alone (−3.0%; 95% CI, −5.3% to −0.8%; *P* = .008) but not including premiums (0.4%; 95% CI, –2.8% to 4.5%; *P* = .82).

**Conclusions and Relevance:**

In this cross-sectional study, US patients with cancer faced significant annual risks of insurance churn and catastrophic health spending. Despite some improvements with ACA implementation, large burdens remained, and further reform is needed to protect this population from excessive hardship.

## Key Points

**Question:**

Was the Patient Protection and Affordable Care Act (ACA) associated with reductions in risks of insurance churn (ie, gain, loss, or change in coverage) and catastrophic health expenditures for nonelderly patients with cancer in the United States?

**Findings:**

In this cross-sectional study, patients with cancer had a lower risk of insurance churn but a higher risk of catastrophic spending vs those without cancer. Implementation of the ACA was associated with small reductions in annual risks of uninsurance and catastrophic spending, although only when excluding premiums.

**Meaning:**

The ACA was associated with small improvements in insurance continuity and out-of-pocket cost burden, but further action is still needed to lessen financial burdens on US patients with cancer and their families.

## Teaser

This cross-sectional study describes the risks of insurance churn (ie, gain, loss, or change in coverage) and catastrophic health expenditures among nonelderly patients with cancer in the US, assessing for changes associated with implementation of the Patient Protection and Affordable Care Act.

## Introduction

Patients with cancer are vulnerable to high out-of-pocket spending, largely owing to frequent interactions with the medical system and high drug prices,^1,2,3,4^ with uninsured individuals at particular risk of financial catastrophe.^5,6^ Insurance coverage is often studied as a static variable, as reported in databases, usually at the time of diagnosis.^7,8^ However, coverage for nonelderly individuals in the United States is dynamic, and insurance churn (ie, gain, loss, or change in coverage) is present even in times of near-universal access to coverage, such as during pregnancy.^9,10,11,12^ Receiving a diagnosis of cancer has been associated with employment disruptions that may affect both income and insurance coverage,^13^ yet we have limited understanding of insurance dynamics and financial burden in this population.

The Patient Protection and Affordable Care Act (ACA) was passed in 2010 with the aim of reducing patients’ financial burden, primarily through improving access to insurance coverage in the US.^14,15^ Patients in the US with cancer were particularly targeted by protections against discrimination on preexisting conditions and new regulations to prevent catastrophic out-of-pocket health spending, both of which took effect soon after the law was signed.^16^ In addition, coverage access improvements through Medicaid expansion and state exchanges began in January 2014.^17^ The ACA had the potential to improve coverage continuity and reduce financial burden for patients in the US with cancer.

The primary objective of this study was to describe the risks of insurance churn and catastrophic health expenditures (CHEs; health expenses exceeding a specified portion of income) among nonelderly patients with cancer in the US. We sought to identify subgroups at high risk, and to assess for changes associated with ACA implementation.

## Methods

### Study Data

We performed a retrospective cross-sectional study of the Medical Expenditure Panel Survey (MEPS), a serial cross-sectional survey encompassing families, medical providers, and insurers, from the Agency for Healthcare Research and Quality.^18,19^ It includes approximately 13 000 families and 30 000 individuals annually, and provides survey weights to extrapolate estimates to the civilian, noninstitutionalized US population (eMethods in the Supplement). All data are deidentified and publicly available, and therefore considered exempt by the Duke Institutional Review Board. In order to assess for changes in association with the ACA, we included respondents to the 2005-2018 surveys, encompassing years prior to passage, (pre-ACA years 2005-2009), during implementation (partial-ACA period, 2010-2013), and after the implementation of all major policies (full-ACA period, 2014-2018). Respondents aged 65 years or older were excluded owing to near universal coverage by Medicare. This study followed the Strengthening the Reporting of Observational Studies in Epidemiology (STROBE) reporting guideline.

### Cancer Diagnosis

Medical conditions are reported in MEPS as self-report of ever receiving a diagnosis of certain priority conditions, including cancer, and in Medical Conditions files with data on health care use and expenditures for the respondent in the given year, by *International Classification of Diseases* code. Patients with cancer were identified as respondents with health care use tied to a cancer diagnosis by *International Classification of Diseases *code in a given year (excluding nonmelanoma skin cancers), as reported in Medical Conditions files (eMethods in the Supplement). Respondents with a self-reported history of cancer in the absence of any associated health care use in that year were excluded. MEPS lacks clinical details, precluding temporal analysis by initial diagnosis or comparisons by cancer treatments.

### Demographic Characteristics

We considered demographic characteristics reported in MEPS that might be associated with health care spending and insurance coverage, including age, sex, race (White, Black, and other or mixed), Hispanic ethnicity, immigrant status, low educational level (no high school degree or equivalent for those older than 18 years), job change (any job change or loss during the year), marital status, family size (number of people), family income in association with the federal poverty level (FPL; ≤138% FPL, 139%-250% FPL, 251%-400% FPL, and >400% FPL),^20^ and self-reported comorbidities. Missing data are rare in MEPS and were not imputed (eMethods in the Supplement).

### Outcomes

In MEPS, month-to-month insurance coverage is reported for each insurer, with multiple insurers reported in cases of complementary or supplemental coverage. We a priori selected our primary insurance churn outcome as insurance loss (change from any insurance to uninsurance on a month-to-month basis). As secondary outcomes we considered any month-to-month change in insurance type and uninsurance at any time during the year. We also assessed Medicaid loss (from Medicaid to uninsurance) and Medicaid disruption (from Medicaid to non-Medicaid insurance or uninsurance) for those with at least 1 month of Medicaid coverage.

For assessment of CHE outcomes, MEPS collects out-of-pocket medical expenses associated with inpatient stays, outpatient visits, emergency department visits, and prescription medications, collected through a combination of self-report and provider or insurer report. Out-of-pocket spending on premiums are reported only for private insurance plans. Premiums for Medicaid can be assumed to be zero.^21^ Medicare premiums were imputed (eMethods in the Supplement). All dollar amounts were adjusted for inflation to 2018 US dollars.^22^ Reported family incomes less than $100 were imputed to $100 to avoid negative or undefined ratios.^23,24^ As finances for medical expenditures are typically shared among a family, we considered the burden of annual health expenditures in terms of family spending and family incomes, consistent with prior MEPS studies.^3,20^

Various thresholds of health expenses to income ratios have been used in prior studies for underinsurance and CHE.^3,20,25^ We a priori selected the Commonwealth Fund definition as our primary outcome^26^: out-of-pocket family health expenses, excluding premiums, exceeding 10% of family income. Given the variation in private insurance plan structure that can lead to costs being more heavily weighted in premium spending for some individuals, we also assessed for CHE including out-of-pocket premium expenses.^20^ Last, given uncertainty in the income thresholds, we also considered CHE as 5%, 20%, and 40% of family income.

### Statistical Analysis

Statistical analysis was conducted from July 30, 2020, to January 5, 2021. Survey weights in MEPS were applied to generate estimates applicable to the noninstitutionalized US population for all analyses. We compared the nonelderly populations with and without a diagnosis of cancer. Among those with cancer, we compared outcomes across subgroups by income, race/ethnicity, and insurance status, and over time in association with ACA implementation. Statistical testing of weighted estimates was performed with the adjusted Wald test. When possible to increase sample size and precision of estimation, data were pooled across years.^27^

To assess for changes in annual risk of insurance churn and CHE over time with ACA implementation, we created a weighted multivariable linear regression model, run on each of 4 outcomes: indicators for annual insurance churn (any uninsurance or insurance loss) and CHE (10% threshold, with and without premiums). The primary exposure was year, grouped in association with ACA implementation, with the coefficient for full implementation from 2014 to 2018, relative to the pre-ACA period (2005-2009), as the primary result of interest. We a priori selected variables for inclusion thought to be potentially associated with churn and CHE risk but not impacted by ACA policies (eMethods in the Supplement).

All *P* values were from 2-sided tests and results were deemed statistically significant at *P* < .05. Analyses were conducted with STATA, version 15.1 (StataCorp LLC), using the ‘SVYSET’ package to account for the complex survey design of MEPS with Taylor series linearization for variance estimation.^28^ Figures were created with Microsoft PowerPoint and Excel, version 14.7.7 (Microsoft Corp).

## Results

### Study Sample

We identified a total of 6069 MEPS respondents younger than 65 years reporting cancer in a given year during 2005-2018 (4280 unique individuals owing to sampling over 2 years), extrapolating to an estimated annual mean of 4.78 million nonelderly patients (95% CI, 4.55-5.01 million; female patients: weighted mean, 63.9% [95% CI, 62.2%-65.7%]; mean age, 50.3 years [95% CI, 49.7-50.8 years]) in the United States with health care use associated with a cancer diagnosis in the given year. The parallel population without cancer in MEPS included 413 314 respondents, representing 261 million individuals in the US annually (eFigure 1 in the Supplement).

### Sample Demographic Characteristics

In comparing the demographic characteristics of the nonelderly population with cancer (Table 1) in the pre-ACA period (2005-2009) with the full ACA years (2014-2018), the population with cancer became 2 years older on average (48.9 years [95% CI, 48.1-49.7 years] vs 50.9 years [95% CI, 50.1-51.6 years]; *P* < .001) and had an increasing proportion of Hispanic patients (6.8% [95% CI, 5.5%-8.0%] vs 9.6% [95% CI, 7.9%-11.2%]; *P* = .005). Relative to those without cancer (eTable 1 in the Supplement), nonelderly patients with cancer were estimated to be older (50.3 years [95% CI, 49.7-50.8 years] vs 30.7 years [95% CI, 30.5-30.9 years]), higher proportion female (63.9% [95% CI, 62.2%-65.7%] vs 49.7% [95% CI, 49.5%-49.9%]), higher proportion white (84.7% [95% CI, 83.3%-86.1%] vs 77.3% [95% CI, 76.1%-78.6%]), lower proportion Hispanic (8.4% [95% CI, 7.3%-9.4%] vs 18.5% [95% CI, 17.2%-19.9%]), and relatively overrepresented in the highest income bracket (>400% FPL; 49.3% [95% CI, 47.1%-51.6%] vs 38.1% [95% CI, 37.2%-38.9%]). Represented cancer sites were generally stable over time and observed changes may reflect data reporting artifacts. To protect confidentiality, more cancers were grouped as “other” in some years by MEPS, particularly in 2016-2018 after the change to the *International Statistical Classification of Diseases and Related Health Problems, Tenth Revision*. We also observed a decrease from 2005-2009 to 2014-2018 in the proportion of patients with urologic or male genital cancers (15.3% [95% CI, 12.8%-17.7%] vs 11.7% [95% CI, 9.9%-13.5%]), contemporaneous with guideline changes that reduced prostate cancer diagnoses (Table 1).^29^

**Table 1.** Demographic Characteristic Estimates for the Nonelderly (<65 Years) Cancer Population in the United States Relative to ACA Implementation, Medical Expenditure Panel Survey, 2005-2018^a^

Characteristic	Weighted mean (95% CI)			*P* value^b^
	Before ACA (2005-2009)	Partial ACA (2010-2013)	Full ACA (2014-2018)	
Sample No. (unweighted)	2052	1850	2167	NA
Annual population (millions)	4.42 (4.10-4.74)	5.03 (4.59-5.46)	4.94 (4.58-5.31)	.02
Age, y	48.9 (48.1-49.7)	51.0 (50.1-51.8)	50.9 (50.1-51.6)	<.001
Sex, %				
Female	64.1 (61.2-67.1)	63.4 (60.2-66.6)	64.2 (61.2-67.2)	.98
Male	35.9 (32.9-38.8)	36.6 (33.4-39.8)	35.8 (32.8-38.8)	.98
Race, %				
White	85.7 (83.4-87.8)	85.9 (83.7-88.1)	82.9 (80.5-85.2)	.09
Black	8.7 (7.3-10.2)	9.9 (8.0-11.7)	10.0 (8.2-11.9)	.28
Other or mixed^c^	5.6 (3.7-7.4)	4.3 (3.0-5.5)	7.1 (5.5-8.7)	.22
Hispanic ethnicity, %	6.8 (5.5-8.0)	8.7 (6.9-10.5)	9.6 (7.9-11.2)	.005
Immigrant, %	9.0 (6.8-11.2)	7.8 (6.2-9.4)	10.1 (8.5-11.7)	.41
Low educational level, %^d^	10.1 (8.3-11.9)	9.0 (7.1-10.8)	8.5 (6.8-10.1)	.18
Job change, %^d^	14.3 (12.3-16.3)	10.2 (8.2-12.2)	10.1 (8.4-11.8)	.002
Married, %	58.9 (55.6-62.2)	62.7 (59.4-65.9)	60.4 (57.3-63.6)	.47
Family size, No.	2.53 (2.45-2.61)	2.52 (2.42-2.62)	2.56 (2.48-2.63)	.65
Family income				
≤138% FPL	15.1 (13.1-17.0)	20.1 (17.8-22.3)	17.4 (15.2-19.7)	.13
139%-249% FPL	15.6 (13.7-17.5)	16.8 (14.5-19.0)	14.0 (12.0-16.0)	.29
250%-400% FPL	19.8 (17.6-22.0)	18.0 (15.5-20.5)	16.1 (13.9-18.3)	.02
>400% FPL	49.5 (46.4-52.6)	45.2 (41.4-49.0)	52.5 (49.0-56.1)	.19
Comorbidities, No.	1.22 (1.13-1.31)	1.49 (1.38-1.61)	1.43 (1.33-1.52)	.002
Cancer, %				
Gynecologic	10.9 (8.8-12.9)	11.8 (9.6-14.0)	7.6 (5.7-9.4)	.02
Breast	21.0 (18.3-23.8)	21.9 (19.0-24.8)	22.8 (20.1-25.6)	.36
GI or hepatobiliary	8.4 (6.6-10.3)	8.8 (7.0-10.7)	7.3 (5.7-8.9)	.34
Urologic or male genital	15.3 (12.8-17.7)	14.6 (12.1-17.1)	11.7 (9.9-13.5)	.02
Lung	3.3 (2.3-4.3)	3.0 (1.9-4.1)	4.5 (3.1-5.9)	.20
Leukemia or lymphoma	8.9 (7.0-10.7)	5.8 (4.4-7.1)	6.3 (4.7-7.9)	.03
Melanoma	8.0 (6.2-9.7)	9.0 (6.7-11.2)	8.5 (6.5-10.6)	.68
Other	24.3 (21.7-26.8)	25.2 (22.2-28.2)	31.3 (27.8-34.7)	.002

Abbreviations: ACA, Patient Protection and Affordable Care Act; FPL, federal poverty level; GI, gastrointestinal; NA, not applicable.

^a^ Cancer diagnosis by listing as *International Classification of Diseases* code in Medical Conditions supplement, representing diagnosis contributing to health care use in given year, excludes nonmelanoma skin cancers.

^b^ *P* value for comparison of full-ACA period (2014-2018) with pre-ACA period (2005-2009) by adjusted Wald test on weighted estimates.

^c^ Other includes any non-White and non-Black races, specifically assessed as American Indian or Alaska Native, Asian, Native Hawaiian or Pacific Islander, and multiple races.

^d^ Percentages exclude persons younger than 18 years; low educational level indicates no high school degree or equivalent; job change indicates at least 1 job change, gain, or loss in year.

### Insurance Churn and CHE Outcomes

In Table 2, we present outcomes of insurance churn and CHE averaged over 2005-2018. In comparison with the population without cancer, nonelderly patients with cancer had lower risks of insurance loss (5.3% [95% CI, 4.5%-6.1%] vs 7.6% [95% CI, 7.4%-7.8%] per year; *P* < .001) and any uninsurance (14.6% [95% CI, 13.3%-16.0%] vs 24.1% [95% CI, 23.5%-24.7%] per year; *P* < .001). Although only 6.1% of those with cancer (95% CI, 5.2%-7.0%) were uninsured for the entire year on average, 14.6% (95% CI, 13.3%-16.0%) spent at least 1 month uninsured in any given year; 24.1% (95% CI, 23.5%-24.7%) of those without cancer spent at least 1 month uninsured in any given year. Relative to those from higher-income families, patients with cancer from families with income 138% or less of the FPL and 250% or less of the FPL were at higher risk of uninsurance all year (≤138% FPL, 11.8% [95% CI, 9.5%-14.2%]; ≤250% FPL, 10.6% [95% CI, 8.8%-12.4%]), insurance loss (≤138% FPL, 9.2% [95% CI, 7.3%-11.2%]; ≤250% FPL, 8.7% [95% CI, 7.2%-10.1%]; *P* < .001), and any change in insurance (≤138% FPL, 24.6% [95% CI, 21.8%-27.3%]; ≤250% FPL, 22.9% [95% CI, 20.9%-24.9%]; *P* < .001). A total of 23.9% (95% CI, 21.4%-26.3%) of those with cancer and income 250% or less than the FPL spent at least 1 month uninsured in any given year.

**Table 2.** Annual Insurance Churn and Catastrophic Health Expenditure Risk Estimates for Nonelderly Patients With Cancer, Medical Expenditures Panel Survey, 2005-2018

Outcome	Weighted mean (95% CI)		*P* value		Cancer diagnosis within subgroups		
	No cancer diagnosis	Cancer diagnosis^a^		≤138% FPL	≤250% FPL	Non-White/Hispanic^b^	Any uninsurance
Sample No.	413 314	6069	NA	1510	2618	2401	1063
Annual estimated No. (millions)	261	4.78	<.001	0.83	1.57	1.10	0.67
Insurance churn							
Insurance loss, %	7.6 (7.4-7.8)	5.3 (4.5-6.1)	<.001	9.2 (7.3-11.2)	8.7 (7.2-10.1)	6.7 (5.4-7.9)	55.8 (50.8-60.8)
Medicaid loss, %	11.5 (11.1-12.0)	9.5 (7.5-11.6)	.06	9.2 (6.5-11.8)	10.0 (7.7-12.3)	9.8 (7.2-12.3)	52.2 (44.1-60.3)
Insurance change, %	15.5 (15.3-15.8)	16.1 (14.9-17.3)	.35	24.6 (21.8-27.3)	22.9 (20.9-24.9)	18.9 (16.6-20.8)	59.7 (55.2-64.2)
Medicaid disruption, %	17.1 (16.6-17.6)	17.7 (15.3-20.1)	.63	14.9 (11.6-18.2)	16.9 (14.2-19.6)	15.7 (12.9-18.5)	53.7 (45.7-61.8)
Any uninsurance, %	24.1 (23.5-24.7)	14.6 (13.3-16.0)	<.001	26.3 (23.2-29.5)	23.9 (21.4-26.3)	18.1 (15.8-20.3)	100 (100-100)
Uninsured all year, %	12.8 (12.3-13.3)	6.1 (5.2-7.0)	<.001	11.8 (9.5-14.2)	10.6 (8.8-12.4)	7.3 (5.9-8.8)	41.6 (37.2-46.1)
**Catastrophic health expenditures**								
Expenses to income ratio, %^c^							
>40	1.9 (1.8-2.0)	3.3 (2.7-3.9)	<.001	15.9 (13.1-18.7)	9.4 (7.6-11.1)	3.8 (2.9-4.7)	6.8 (4.9-8.7)
>20	3.2 (3.1-3.3)	6.1 (5.2-6.9)	<.001	24.4 (21.1-27.6)	16.1 (13.9-18.3)	6.5 (5.3-7.6)	11.1 (8.7-13.5)
>10	6.3 (6.2-6.5)	12.4 (11.2-13.6)	<.001	36.0 (32.3-39.6)	29.1 (26.4-31.8)	11.2 (9.7-12.8)	20.6 (17.3-24.0)
>5	14.0 (13.7-14.3)	24.7 (23.0-26.4)	<.001	48.9 (45.2-52.6)	43.3 (40.4-46.3)	21.7 (19.5-23.8)	36.5 (32.1-40.9)
Expenses plus premiums to income ratio, %^c^							
>40	2.6 (2.5-2.8)	5.0 (4.2-5.7)	<.001	22.2 (19.0-25.4)	14.0 (12.0-16.0)	4.9 (3.9-5.9)	7.8 (5.7-9.9)
>20	5.9 (5.8-6.1)	11.6 (10.5-12.6)	<.001	33.8 (30.1-37.5)	26.5 (24.0-29.0)	9.8 (8.4-11.2)	14.1 (11.4-16.8)
>10	16.5 (16.1-16.8)	26.6 (25.0-28.1)	<.001	48.3 (44.3-52.3)	45.4 (42.5-48.3)	21.9 (19.8-24.1)	27.3 (23.5-31.1)
>5	37.7 (37.1-38.3)	51.9 (50.1-53.6)	<.001	60.3 (56.6-64.0)	61.8 (59.0-64.5)	44.2 (41.2-47.1)	49.9 (45.2-54.6)

Abbreviations: FPL, federal poverty level; NA, not applicable.

^a^ Cancer diagnosis by listing in Medical Conditions files as *International Classification of Diseases* code contributing to medical care in given year; excludes nonmelanoma skin cancers.

^b^ Includes persons of nonwhite race and/or Hispanic ethnicity.

^c^ Ratio of family level out-of-pocket expenses (for health care services) with or without family insurance premiums to family income.

For our primary CHE outcome of family health care out-of-pocket expenses, excluding premiums, of more than 10% of family income, we found 12.4% (95% CI, 11.2%-13.6%) of those with cancer reported CHE annually by expenses alone, compared with 6.3% (95% CI, 6.2%-6.5%) of those without cancer (Table 2). When including premium spending, the risk of CHE was 26.6% (95% CI, 25.0%-28.1%) among those with cancer, compared with 16.5% (95% CI, 16.1%-16.8%) among those without cancer. Relative to those without cancer, patients with cancer faced approximately 1.5 to 2 times the risk of CHE, irrespective of the threshold chosen, or inclusion or exclusion of premiums. Those with cancer in low-income families faced higher risks of CHE across all considered outcomes. Among those with cancer and family income of 250% or less than the FPL, 29.1% (95% CI, 26.4%-31.8%) reported CHE by expenses alone and 45.4% (95% CI, 42.5%-48.3%) reported CHE by expenditures plus premiums. A lower threshold for CHE among poor and near-poor families has been proposed.^20^ Family health spending exceeded 5% of family income, including premium spending, for 61.8% (95% CI, 59.0%-64.5%) of those with cancer in families with income of 250% or less than the FPL.

Catastrophic health spending among nonelderly US patients with cancer was not limited to those with fragmented coverage or periods of uninsurance, but included those with stable full-year private insurance or Medicaid coverage (Table 3). By expenses alone at the primary threshold of 10%, those with full-year Medicaid coverage faced a greater risk of CHE relative to those with full-year private coverage (14.1% [95% CI, 11.2%-17.0%] vs 9.0% [95% CI, 7.7%-10.2%]; *P* = .001). However, when incorporating premium spending, those with Medicaid faced a lower risk of CHE vs those with private coverage (17.4% [95% CI, 14.3%-20.5%] vs 25.9% [95% CI, 24.1%-27.8%]; *P* < .001). Furthermore, low-income families with full-year private coverage faced a particularly high risk of CHE, with 81.7% (95% CI, 74.6%-88.9%) of those from families with income of 138% of less than the FPL and 64.7% (95% CI, 60.1%-69.3%) of those from families with income of 250% or less than the FPL reporting CHE (at the 5% threshold: ≤138% FPL, 92.2% [95% CI, 88.5%-95.9%]; ≤250% FPL, 82.2% [95% CI, 78.8%-85.6%]).

**Table 3.** Catastrophic Health Expenditure Risk Estimates for Nonelderly Patients With Cancer With Stable Full-Year Coverage, Private vs Medicaid Coverage, Medical Expenditures Panel Survey, 2005-2018

Outcome	Full-year private coverage			Full-year Medicaid coverage			*P* values		
	All	≤138% FPL	≤250% FPL	All	≤138% FPL	≤250% FPL	All	≤138% FPL	≤250% FPL
Sample No.	3496	224	725	985	672	870	NA	NA	NA
Annual estimated No. (millions)	3.23	0.17	0.55	0.50	0.32	0.43	<.001	.001	.04
Expenses to income ratio, %^a^									
>40	1.8 (1.2-2.3)	22.2 (15.6-28.8)	9.0 (6.2-11.7)	5.7 (3.8-7.6)	8.9 (5.8-11.8)	6.4 (4.2-8.7)	.002	<.001	.13
>20	4.0 (3.1-4.9)	38.3 (30.1-46.5)	18.2 (14.3-22.1)	9.5 (7.0-11.9)	13.9 (10.2-17.6)	10.5 (7.7-13.4)	<.001	<.001	.002
>10	9.0 (7.7-10.2)	57.7 (49.2-66.2)	34.5 (30.0-38.9)	14.1 (11.2-17.0)	17.3 (13.4-21.1)	15.3 (11.9-18.6)	.001	<.001	<.001
>5	20.6 (18.7-22.5)	74.2 (66.6-81.8)	51.4 (46.3-56.4)	23.7 (20.1-27.4)	27.4 (22.7-32.0)	24.4 (20.4-28.4)	.18	<.001	<.001
Expenses plus premiums to income ratio, %^a^									
>40	3.7 (2.9-4.5)	44.2 (35.8-52.6)	18.9 (15.0-22.8)	6.3 (4.3-8.3)	9.3 (6.3-12.3)	6.8 (4.6-9.1)	.05	<.001	<.001
>20	10.1 (8.8-11.3)	62.6 (54.0-71.1)	38.4 (33.8-43.0)	10.5 (7.9-13.1)	14.6 (10.8-18.3)	11.7 (8.7-14.7)	.93	<.001	<.001
>10	25.9 (24.1-27.8)	81.7 (74.6-88.9)	64.7 (60.1-69.3)	17.4 (14.3-20.5)	19.6 (15.7-23.5)	17.7 (14.3-21.1)	<.001	<.001	<.001
>5	53.8 (51.5-56.0)	92.2 (88.5-95.9)	82.2 (78.8-85.6)	30.6 (26.4-34.7)	31.2 (26.2-36.2)	29.4 (25.0-33.7)	<.001	<.001	<.001

Abbreviations: FPL, federal poverty level; NA, not applicable.

^a^ Catastrophic health expenditure by ratio of family level out-of-pocket expenses (for health care services) with or without family private insurance premiums to family income; cancer diagnosis by listing as *International Classification of Diseases* code contributing to medical care in given year; excludes nonmelanoma skin cancers.

### Trends Over Time With ACA Implementation

**Figure 1.**
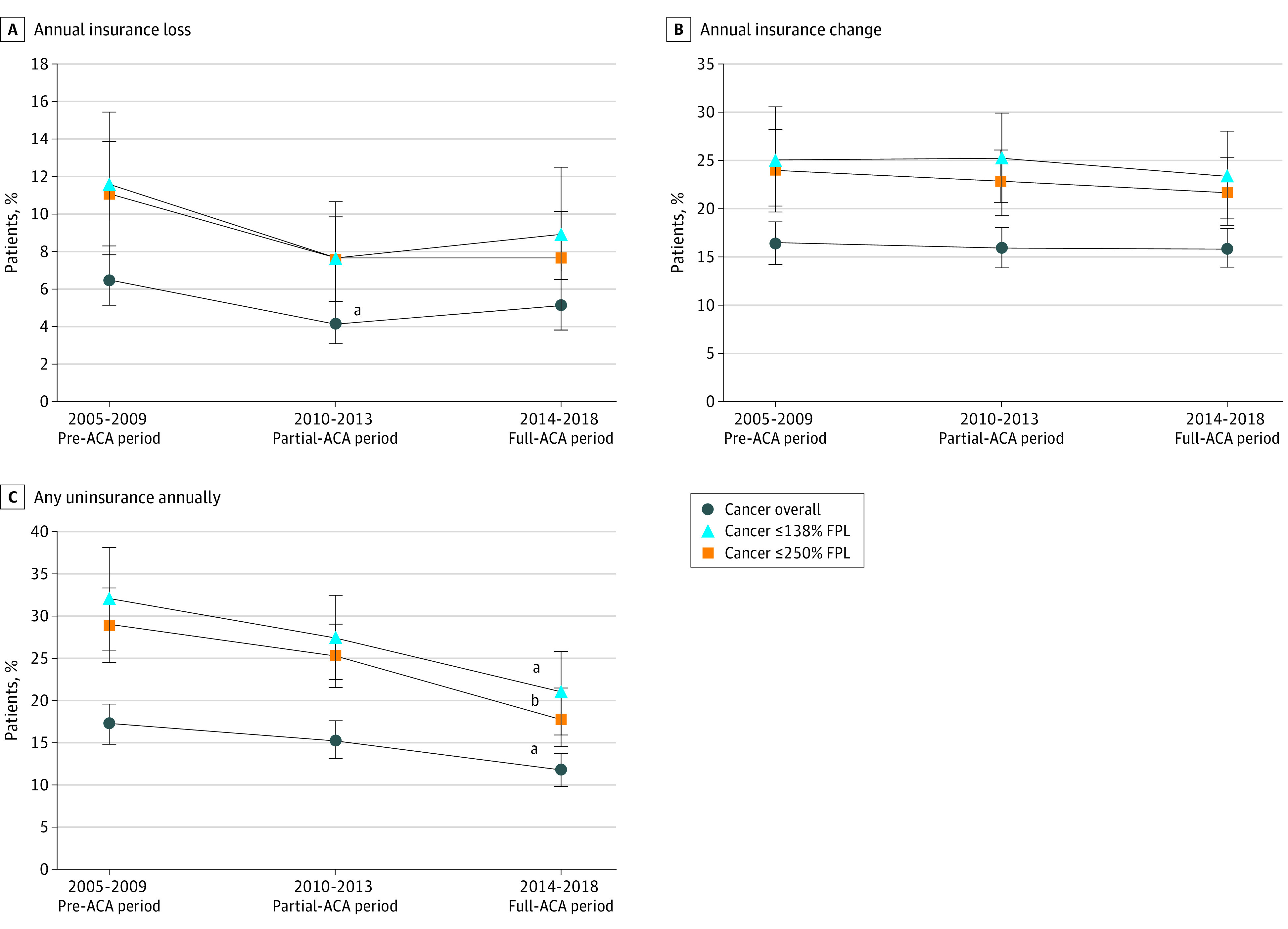
Time Trends in Annual Risks of Insurance Churn Outcomes Pooled According to Time Relative to Patient Protection and Affordable Care Act (ACA) Implementation Among Nonelderly Patients With Cancer, Medical Expenditure Panel Survey, 2005-2018 A, Annual insurance loss. B, Annual insurance change. C, Any uninsurance annually. FPL indicates federal poverty level. Error bars indicate 95% CIs. ^a^*P* < .01 relative to 2005-2009 pre-ACA period. ^b^*P* < .001 relative to 2005-2009 pre-ACA period.

**Figure 2.**
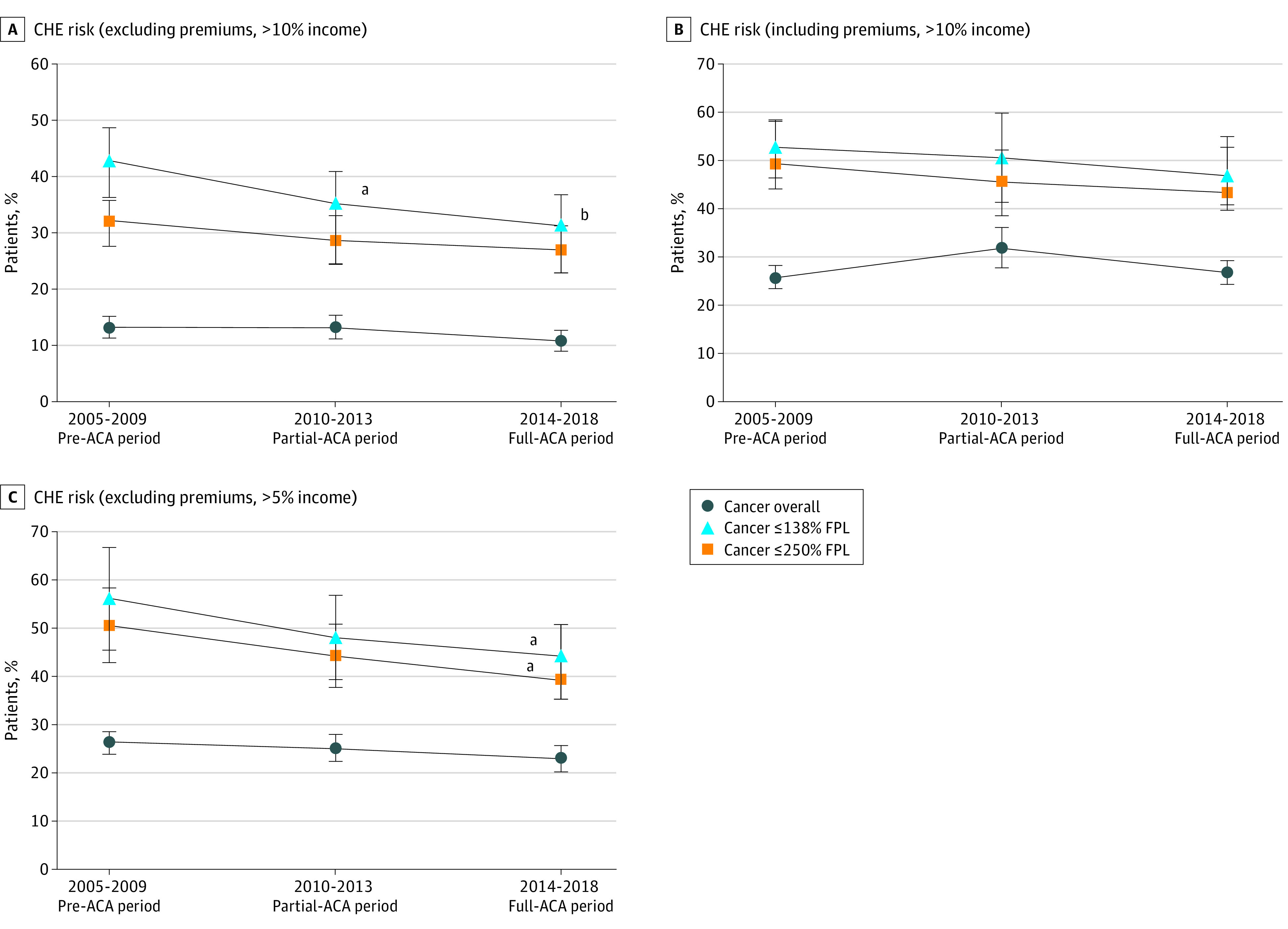
Time Trends in Annual Risks of Catastrophic Health Expenditures (CHE) Pooled According to Time Relative to Patient Protection and Affordable Care Act (ACA) Implementation Among Nonelderly Patients With Cancer, Medical Expenditure Panel Survey, 2005-2018 A, CHE risk (excluding insurance premiums, >10% income). B, CHE risk (including insurance premiums, >10% income). C, CHE risk (excluding insurance premiums, >5% income). FPL indicates federal poverty level. Error bars indicate 95% CIs. ^a^*P* < .05 relative to 2005-2009 pre-ACA period. ^b^*P* < .01 relative to 2005-2009 pre-ACA period.

We first assessed raw time trends associated with ACA implementation for nonelderly patients with cancer in Figure 1 and Figure 2 (see eFigure 2 and eFigure 3 in the Supplement for comparison with patients without cancer). Among nonelderly patients with cancer, we found generally stable risks of annual insurance change of any kind, but steady declines in annual risk of any uninsurance, with lower risks after the ACA was fully implemented (2014-2018, 11.8% [95% CI, 9.8%-13.8%] vs 2005-2009, 17.2% [95% CI, 14.8%-19.5%]; *P* = .001), particularly among those with a family income of 250% or less than the FPL (18.0% [95% CI, 14.5%-21.4%] vs 29.0% [95% CI, 24.5%-33.5%]; *P* < .001). Relative to the population without cancer, those with cancer had similar estimated declines in the risks of CHE excluding premiums, but reductions in CHE reached statistical significance only among low-income subgroups for patients with cancer. When including premium spending, there were no statistically significant declines in CHE with ACA implementation, regardless of income group.

We used multivariable regression to assess for the association of insurance churn (any uninsurance or insurance loss; eTable 2 in the Supplement) and CHE (spending >10% of income, with and without premiums; eTable 3 in the Supplement) with ACA implementation among patients with cancer. With adjustment, full ACA implementation (2014-2018) was associated with decreased absolute risk of any uninsurance (−4.2%; 95% CI, −7.4% to −1.0%; *P* = .01) and decreased risk of CHE excluding premiums (−3.0%; 95% CI, −5.3% to 0.8%; *P* = .008), relative to the pre-ACA period (2005-2009) among patients with cancer. However, there was no change in risks of insurance loss (−0.9%; 95% CI, –2.8% to 1.1%; *P* = .36), or CHE including premiums (0.4%; 95% CI, –2.8% to 3.5%; *P* = .82). The primary observed benefit associated with the ACA for nonelderly US patients with cancer was in gains in insurance coverage associated with lower out-of-pocket spending. However, when premium spending was included, we did not find any reductions associated with ACA implementation.

In assessing the association of other included covariates, we found income to be the factor associated most strongly with risk in all models. Relative to incomes greater than 400% of the FPL, those with income of 138% or less than the FPL faced 6.5% (95% CI, 4.2%-8.8%; *P* < .001) increased annual risk of insurance loss, 17.3% (95% CI, 13.4%-21.2%; *P* < .001) increased annual risk of any uninsurance, and 37.4% (95% CI, 33.3%-41.6%; *P* < .001) increased annual risk of CHE exceeding 10% of income. We also found job change or loss to be strongly associated with increased risk (10.6% [95% CI, 6.7%-14.3%] increased annual risk of insurance loss and 15.7% [95% CI, 10.9%-20.5%] increased annual risk of any uninsurance). Presence of a second major comorbidity in addition to cancer was associated with decreased risks of churn (insurance loss, −1.8% [95% CI, –3.2% to –0.4%]; and any uninsurance, –4.1% [95% CI, –6.9% to –1.2%]), but similar risks of CHE (excluding premiums, 1.9% [95% CI, –1.0% to 4.7%; and including premiums, 3.1% [95% CI, –0.5% to 6.8%]). Despite higher risk of any uninsurance among Hispanic patients with cancer (5.4% [95% CI; 1.4%-9.4%]), they faced lower risks of CHE relative to non-Hispanic patients (−5.1% [95% CI, −7.9% to −2.3%).

## Discussion

Our primary aim was to describe insurance churn and CHE among nonelderly patients with cancer and assess for potential improvements associated with ACA implementation. We found that nonelderly US patients with health care use associated with a cancer diagnosis in a given year were at overall lower risk of insurance churn than the population without cancer, likely owing to the strong incentive to maintain continuous insurance coverage. Despite higher rates of coverage, those with cancer faced far higher risks of CHE, likely owing to increased health care use.The policy reforms of the ACA were associated with limited improvements in these outcomes.

Among nonelderly patients with cancer, full ACA implementation was associated with a 4% absolute reduction in risk of any uninsurance annually. Although insurance churn has been assessed previously in other populations,^11,12^ our study is novel in its consideration of the longitudinal dynamics of insurance coverage in the population of patients with cancer specifically. Most database studies report coverage at diagnosis and apply this coverage status to the duration of a patient’s care, with one finding reduced uninsurance at cancer diagnosis from 5.7% to 3.8% after full ACA implementation.^30^ However, these static numbers undersell the extent of underinsurance over a period of time. We found that 10% to 14% of all patients with cancer, and 16% to 26% of those with family income of 138% or less than the FPL were uninsured for at least 1 month in any given year, even with full ACA implementation.

Full ACA implementation was also associated with 3% lower risk of CHE by expenses alone for patients with cancer. However, we found no associated reductions in CHE when including spending on insurance premiums, despite the fact that we assumed zero premiums for Medicaid coverage. Furthermore, patients from low-income families were at higher risk of CHE with private insurance coverage vs Medicaid, in agreement with a recent study of a noncancer population.^31^ Underinsurance owing to high premiums likely counterbalanced cost savings from gaining private insurance coverage. It seems that low-income patients with cancer are better protected from CHE by Medicaid coverage than by private insurance coverage. With the exception of the Medicaid expansion, which was not adopted in all states, most ACA policies targeted the expansion of private insurance coverage.

Our study builds on the literature regarding the financial burden of cancer care. In a pre-ACA study using MEPS 2001-2008, authors used similar methods and found that 13.4% of those with cancer used more than 20% of income for out-of-pocket expenses and premiums,^3^ which is higher than the 11.6% we observe in the ACA era from 2010-2018. Another MEPS study of respondents with cancer from 2011-2015 identified nonsignificant trends in reduced spending with the ACA.^32^ We build on this work by including a larger sample from more ACA years, and considering insurance churn outcomes and the interplay with financial burden. A nationally representative survey of persons aged 50 years or older with cancer found that more than 40% reported complete asset depletion within 2 years of diagnosis.^5^ Such financial burden for patients has been associated with decreased quality of life^33^ and foregoing recommended care.^2,4^

### Strengths and Limitations

This study has some strengths. The main strength is in leveraging advantages of MEPS to form a representative description of nonelderly patients with cancer in the US. We used MEPS survey weights to generate estimates applicable to the civilian, noninstitutionalized US population. Our definition of cancer was designed to capture those receiving care for cancer that may be associated with insurance-seeking and health care use behavior. Unlike some surveys, MEPS assesses providers and insurers to verify health care use and expenditures rather than relying exclusively on patient self-report. Furthermore, as families typically share medical expenses, we leveraged family identification in MEPS to calculate CHE as the ratio of family expenditures to family income, consistent with prior studies.

This study also has some limitations. Our primary limitation was the relatively small sample size of the nonelderly sample with cancer, which limited the precision of our estimates, including our ability to detect significant differences for small changes over time associated with the ACA. MEPS medical condition data offer limited detail on cancer type and no information on the cancer course, cancer stage, or treatment of the individuals under study. *International Classification of Diseases* code grouping was not entirely consistent year to year in MEPS, limiting the ability to compare different cancer sites. MEPS contains no quantifiable information on nonmedical costs of the cancer diagnosis, such as transportation and parking^34^ and opportunity cost from lost or limited employment. State of residence is not reported in MEPS, and we were therefore unable to isolate the association of Medicaid expansion uptake by a subset of states independent of other ACA policies.

## Conclusions

Nonelderly patients with cancer in the US continue to cope with a complex insurance marketplace and high risks of CHE, despite the well-intentioned policy reforms of the ACA. In particular, low-income patients with cancer with private insurance coverage may face lower risks of periods of uninsurance, but underinsurance is associated with high risks of CHE. Oncologists should be cognizant of the financial strains that cancer places on patients and families, even when insured. Further health reforms to cover the remaining uninsured individuals and to increase plan generosity are needed to adequately protect the population of individuals with cancer, which already faces significant physical and mental burden from disease.

## Notes

Supplement.
eMethods.
**eTable 1.** Demographic Characteristic Estimates for the Nonelderly (<65) Population Without Cancer in the United States Relative to ACA Implementation, Medical Expenditure Panel Survey, 2005-2018 **eTable 2.** Weighted Multivariable Linear Regression Models for Association of Affordable Care Act With Annual Risk of Insurance Churn (Insurance Loss, Any Uninsurance) Among the Nonelderly (<65) Cancer Population, Medical Expenditures Panel Survey, 2005-2018 **eTable 3.** Weighted Linear Regression Models for Predictors of Catastrophic Health Expenditures (Family Expenses >10% Family Income) Among the Nonelderly (<65) Cancer Population, Medical Expenditures Panel Survey, 2005-2018 **eFigure 1.** CONSORT Diagram of Data Management and Study Population Selection for Study of Patients With Cancer Relative to Those Without Cancer, Medical Expenditure Panel Survey, 2005-18 **eFigure 2.** Trends in Insurance Churn Risks for the Nonelderly Noncancer Population, Relative to the Implementation of the Affordable Care Act, Medical Expenditure Panel Survey, 2005-2018 **eFigure 3.** Trends in Catastrophic Health Expenditures (Ratio of Family Out-of-Pocket Expenses to Family Income) Risks for the Nonelderly Noncancer Population, Relative to the Implementation of the Affordable Care Act, Medical Expenditure Panel Survey, 2005-2018
