# Supplementary material for: Associations of Insurance Churn and Catastrophic Health Expenditures With Implementation of the Affordable Care Act Among Nonelderly Patients With Cancer in the United States
Source: JAMA Netw Open. 2021 Sep 8;4(9):e2124280. doi: 10.1001/jamanetworkopen.2021.24280 (PMC8427370; doi:10.1001/jamanetworkopen.2021.24280)
Supplement: Supplement. — eMethods. eTable 1. Demographic Characteristic Estimates for the Nonelderly (<65) Population Without Cancer in the United States Relative to ACA Implementation, Medical Expenditure Panel Survey, 2005-2018 eTable 2. Weighted Multivariable Linear Regression Models for Association of Affordable Care Act With Annual Risk of Insurance Churn (Insurance Loss, Any Uninsurance) Among the Nonelderly (<65) Cancer Population, Medical Expenditures Panel Survey, 2005-2018 eTable 3. Weighted Linear Regression Models for Predictors of Catastrophic Health Expenditures (Family Expenses >10% Family Income) Among the Nonelderly (<65) Cancer Population, Medical Expenditures Panel Survey, 2005-2018 eFigure 1. CONSORT Diagram of Data Management and Study Population Selection for Study of Patients With Cancer Relative to Those Without Cancer, Medical Expenditure Panel Survey, 2005-18 eFigure 2. Trends in Insurance Churn Risks for the Nonelderly Noncancer Population, Relative to the Implementation of the Affordable Care Act, Medical Expenditure Panel Survey, 2005-2018 eFigure 3. Trends in Catastrophic Health Expenditures (Ratio of Family Out-of-Pocket Expenses to Family Income) Risks for the Nonelderly Noncancer Population, Relative to the Implementation of the Affordable Care Act, Medical Expenditure Panel Survey, 2005-2018 [file jamanetwopen-e2124280-s001.pdf]

Supplementary Online Content

Albright BB, Chino F, Chino JP, Havrilesky LJ, Aviki EM, Moss HA. Associations of insurance churn and catastrophic health expenditures with implementation of the Affordable Care Act among nonelderly patients with cancer in the United States. *JAMA Netw Open.* 2021;4(9):e2124280.
doi:10.1001/jamanetworkopen.2021.24280

eMethods.
eTable 1. Demographic Characteristic Estimates for the Nonelderly (<65) Population Without Cancer in the United States Relative to ACA Implementation, Medical Expenditure Panel Survey, 2005-2018
eTable 2. Weighted Multivariable Linear Regression Models for Association of Affordable Care Act With Annual Risk of Insurance Churn (Insurance Loss, Any Uninsurance) Among the Nonelderly (<65) Cancer Population, Medical Expenditures Panel Survey, 2005-2018
eTable 3. Weighted Linear Regression Models for Predictors of Catastrophic Health Expenditures (Family Expenses >10% Family Income) Among the Nonelderly (<65) Cancer Population, Medical Expenditures Panel Survey, 2005-2018
eFigure 1. CONSORT Diagram of Data Management and Study Population Selection for Study of Patients With Cancer Relative to Those Without Cancer, Medical Expenditure Panel Survey, 2005-18
eFigure 2. Trends in Insurance Churn Risks for the Nonelderly Noncancer Population, Relative to the Implementation of the Affordable Care Act, Medical Expenditure Panel Survey, 2005-2018
eFigure 3. Trends in Catastrophic Health Expenditures (Ratio of Family Out-of-Pocket Expenses to Family Income) Risks for the Nonelderly Noncancer Population, Relative to the Implementation of the Affordable Care Act, Medical Expenditure Panel Survey, 2005-2018

This supplementary material has been provided by the authors to give readers additional information about their work.

© 2021 Albright BB et al. *JAMA Network Open.*

eMethods

Study Data
The Medical Expenditure Panel Survey represents a subsample of the prior year's National health Interview Survey. It includes intentional oversampling of Black and Asian race respondents, and Hispanic ethnicity respondents. Due to MEPS sampling procedures, some individuals are represented twice in the data as an observation in two consecutive years. Response rates for MEPS declined over time during the years under study, from a peak of 59.3% in 2008 to 44.2% in 2017.

We utilized MEPS Household Component 'Full Year Consolidated', 'Medical Condition', and 'Person Round Plan' files. MEPS data are largely complete, with internal imputation procedures performed for missing data for certain variables. No further data imputation procedures were performed.

Cancer Diagnosis
For years 2005 to 2015, we identified cancer diagnoses by ICD-9 Clinical Classifications Software codes (11-22; 24-43). For years 2016 to 2018, we identified cancer diagnoses by ICD-10 codes (C00-C43; C45-C97; D49). Cancer diagnoses were grouped by primary site (gynecologic, breast, gastrointestinal/hepatobiliary, urologic/male genital, lung, leukemia/lymphoma, melanoma, and other). In some cases, the reported codes were simplified or grouped to avoid samples considered too small for accurate estimation. Reported cancer sites varied year-to-year, with those not reported individually grouped into an 'other' category. MEPS contains limited clinical details on the care utilized in reference to medical conditions. It was not possible to determine the date of diagnosis, or type of cancer care being received, whether it be surgery, radiation, chemotherapy, surveillance, or palliative care.

Self-reported history of ever receiving a cancer diagnosis was collected as priority condition information in MEPS starting in 2008. Respondents with such a self-reported historical diagnosis in absence of a current diagnosis by ICD code utilization in the last year were not considered current cancer patients. While not used to assign a current cancer diagnosis, self-reported cancer history was used to assign the cancer site when cancer diagnosis was coded as 'other' with the site not specified by ICD codes. Respondents who self-reported a history of a cancer diagnosis in absence of any associated utilization in the last year were not included among the considered cancer population, as we sought to limit to cases requiring ongoing care that might affect spending and coverage-seeking behavior.

Demographics
Demographic survey data was utilized from the MEPS Full-Year Consolidated files. As date of cancer diagnosis and phase of treatment were unknown and cancer diagnosis was assessed by healthcare utilization in a given year, survey responses to demographic questions may have, in some cases, preceded the cancer diagnosis.

Income in relation to federal poverty level (FPL) is provided within MEPS. The particularly utilized categorization (≤138% FPL, 139-250% FPL, 251-400% FPL, >400% FPL) was selected *a priori* in concordance with prior studies of the ACA (Goldman, *et al.* 2018, Gotanda, *et al.* 2020) in relation to the Federal Poverty Level (FPL), with thresholds selected corresponding to ACA policy eligibility thresholds. Medicaid expansion policies extended to those ≤138% FPL, while those between 138% and 250% of the FPL generally qualify for subsidies and reduced cost sharing for state exchange insurance plans, and those between 251% and 400% of FPL generally qualify for subsidies without reduced cost sharing for state exchange plans.

Comorbidities are collected by MEPS via self-report of ever being diagnosed with any of the considered priority conditions, including hypertension, hypercholesterolemia, coronary artery disease, angina, stroke, myocardial infarction, other heart disease, diabetes, emphysema, asthma, and arthritis. We considered comorbidities as a count of total reported diagnoses. In regression analyses, we considered a dichotomous indicator for presence of a major comorbidity (coronary artery disease, myocardial infarction, stroke, diabetes, or emphysema).

As all patients in the primary analytic cohort had a cancer diagnosis, the cancer diagnosis itself was not included in the count of comorbidities or as a major comorbidity. As comorbid diagnoses were not a central focus of this study and ICD codes are not reported in full detail, we did not attempt to impute self-reported diagnoses with healthcare utilization in the last year by ICD codes reported in the Medical condition files.

© 2021 Albright BB et al. *JAMA Network Open.*

Missing data were rare in MEPS and not imputed. Among the cancer cohort, there was complete data for all utilized demographic variables with the exceptions of job change (0.2%), and education (1.0%).

Outcomes
For insurance churn outcomes, we considered public coverage including Tricare/Veteran's Administration, Medicare, Medicaid, and other public coverage, as well as private coverage in any form.

As families generally share the burden of health care expenses, all expenditure outcomes were considered at the family level and applied to individuals. In MEPS, a family is defined as two or more persons living together in the same household who are related by blood, marriage, or adoption, or unmarried persons living together who consider themselves a family unit.

As our analysis is limited to individuals under 65 years old, few of the included individuals have Medicare coverage. However, since we considered expenses at the family level, some individuals under 65 are in the same family as individuals over 65 and covered by Medicare. Therefore, Medicare premiums were imputed for family members with months of Medicare coverage. No premiums were added in the case of concurrent Medicaid coverage or supplemental private coverage, as these supplemental policies typically pay for some or all of the Part B premium. Premiums were imputed at the baseline rate for that year. The complex small increases in Medicare premiums for the wealthy are based on how taxes are filed and are unlikely to contribute significantly to catastrophic health spending.

Definitions for catastrophic health spending and underinsurance vary in the literature, both in proposals from organizations such as the World Health Organization and the Commonwealth Fund, and in utilized thresholds in comparable studies, many of which also use MEPS data. We *a priori* selected the Commonwealth Fund definition as our primary outcome: family out-of-pocket healthcare expenses exceeding 10% of family income. However, given the uncertainty in the definition, we also present alternative definitions. We also assessed for CHE including out-of-pocket premium contributions to account for variation in distribution of costs among privately insured patients. Additionally, we assessed alternative thresholds of 5%, 20% and 40% of income.

Statistical Analysis
Subgroups for comparison (income, race/ethnicity, and insurance status) were selected *a priori* based on interest in identifying potential disparities between groups.

In order to assess for the impact of the Affordable Care Act in changing risk of insurance churn and CHE over time, we created regression models to adjust for other demographic variables that may influence these risks. We considered four total outcomes: insurance churn by any uninsurance or insurance loss dichotomous indicators, and catastrophic health expenditures by 10% threshold, excluding or including premiums. The primary exposure of interest was year in relation to the implementation of the Affordable Care Act. We included other contextual demographic covariates in the models that were felt to be potentially relevant to the outcomes of insurance churn and catastrophic health expenditures: age (<27, 27-44, 45-64), sex, race (white, Black, other/mixed), ethnicity (Hispanic vs. non-Hispanic), self-reported major comorbidity (presence of any of coronary artery disease, myocardial infarction, stroke, diabetes, or emphysema), education (no high school degree or equivalent for age >18), job change during the year, marital status, family size (count of people), family income (≤138% FPL, 139-250% FPL, 251-400% FPL, >400% FPL), and year (relative to Affordable Care Act implementation: 2005-2009, 2010-2013, 2014-2018). Rare observations with missing data were omitted from regressions (1.2% of observations).

Of note, MEPS does not identify the respondent's state of residence, and we were therefore unable to independently assess for the impact of Medicaid expansion, which was adopted as a part of the Affordable Care Act by some, but not all states.

© 2021 Albright BB et al. *JAMA Network Open.*

eTable 1. Demographic Characteristic Estimates for the Nonelderly (<65) Population Without Cancer[a] in the United States Relative to ACA Implementation, Medical Expenditure Panel Survey, 2005-2018

	**Pre ACA 2005-2009** weighted mean (95%CI)	**Partial ACA 2010-2013** weighted mean (95%CI)	**Full ACA 2014-2018** weighted mean (95%CI)	***P* Value[b] Full ACA vs. Pre ACA**	***P* Value[b] Cancer vs. Non-cancer**
**Sample n** (unweighted)	148,214	124,887	140,213	-	-
**Annual Pop.** (millions)	259 (250–267)	261 (250–272)	264 (256–273)	0.25	<0.001
**Age** (years)	30.5 (30.2–30.7)	30.6 (30.3–30.9)	30.9 (30.7–31.2)	0.005	<0.001
**Sex** (%)					
Female	49.7% (49.3–50.0)	49.7% (49.3–50.1)	49.7% (49.4–50.1)	0.79	<0.001
Male	50.3% (50.0–50.7)	50.3% (49.9–50.7)	50.3% (49.9–50.6)	0.79	<0.001
**Race** (%)					
White	79.1% (78.0–80.2)	78.1% (76.3–79.8)	75.1% (73.6–76,5)	<0.001	<0.001
Black	13.1% (12.1–14.0)	13.1% (11.8–14.5)	13.6% (12.5–14.7)	0.28	<0.001
Other/mixed	7.8% (7.2–8.5)	8.8% (7.8–9.8)	11.3% (10.5–12.2)	<0.001	<0.001
**Hispanic ethnicity** (%)	16.8% (15.6–18.0)	18.7% (16.7–20.7)	20.1% (18.6–21.5)	<0.001	<0.001
**Immigrant** (%)	13.3% (12.5–14.1)	13.8% (12.8–14.7)	14.4% (13.6–15.1)	0.003	<0.001
**Low Education[c]** (%)	12.3% (11.8–12.9)	10.7% (10.0–11.4)	9.6% (9.1–10.1)	<0.001	0.001
**Job Change[c]** (%)	16.1% (15.8–16.5)	14.7% (14.4–15.1)	15.7% (15.4–16.1)	0.13	<0.001
**Married** (%)	39.7% (39.2–40.2)	37.7% (37.0–38.4)	37.4% (36.8–37.9)	<0.001	<0.001
**Family Size** (n)	3.4 (3.3–3.4)	3.34 (3.3–3.4)	3.4 (3.3–3.4)	0.98	<0.001
**Family Income** (%)					
<100% FPL	19.5% (18.7–20.2)	22.8% (21.7–24.0)	20.1% (19.3–21.0)	0.14	<0.001
100-200% FPL	19.9% (19.3–20.6)	20.6% (20.0–21.2)	19.5% (18.9–20.1)	0.29	<0.001
200-400% FPL	22.4% (21.8–22.9)	21.0% (20.3–21.6)	20.5% (19.8–21.1)	<0.001	<0.001
>400% FPL	38.2% (37.2–39.2)	35.6% (34.3–36.9)	39.9% (38.8–40.9)	0.01	<0.001
**Comorbidities[d]** (n)	0.51 (0.49–0.51)	0.55 (0.54–0.56)	0.55 (0.54–0.56)	<0.001	<0.001

ACA = Affordable Care Act; weighted annual estimates pooling within indicated time periods
[a] Excludes all respondents with self-reported history of cancer or cancer diagnosis by listing as ICD code in Medical Conditions supplement, representing diagnosis contributing to healthcare utilization in given year, exclusions do not include non-melanoma skin cancer diagnoses
[b] *P* value by adjusted Wald test on weighted estimates in comparison to cancer cohort as defined by listing as ICD code in Medical Conditions supplement

© 2021 Albright BB et al. *JAMA Network Open.*

[c] Percentages exclude persons <18 years old; low education indicates no post-high school degree, job change indicates at least 1 job change, gain, or loss in year
[d] Self-reported count of non-cancer comorbidities (see **eMethods**)

© 2021 Albright BB et al. *JAMA Network Open.*

eTable 2. Weighted Multivariable[a] Linear Regression Models for Association of Affordable Care Act With Annual Risk of Insurance Churn[b] (Insurance Loss, Any Uninsurance) Among the Nonelderly (<65) Cancer Population, Medical Expenditures Panel Survey, 2005-2018

	**Insurance Loss**		**Any Uninsurance**	
	**β (95%CI)**	***P* Value**	**β (95%CI)**	***P* Value**
**Age**				
<27 years	ref.	-	ref.	-
27-44 years	-1.3% (-5.6–3.0)	0.56	-5.0% (-11.8–1.7)	0.14
45-64 years	-3.2% (-7.4–0.9)	0.13	-7.0% (-14.1–0.0)	0.05
**Female Sex**	1.1% (-0.3–2.5)	0.14	-0.2% (-2.4–2.9)	0.86
**Race**				
White	ref.	-	ref.	-
Black	0.3% (-1.8–2.4)	0.79	-1.0% (-4.4–2.5)	0.58
Other	1.7% (-1.4–4.9)	0.27	2.1% (-3.5–7.6)	0.47
**Hispanic Ethnicity**	0.9% (-1.2–3.0)	0.39	5.4% (1.4–9.4)	0.008
**Major comorbidity**[c]	-1.8% (-3.2– -0.4)	0.01	-4.1% (-6.9– -1.2)	0.005
**Low Education**[d]	-0.3% (-2.8–2.2)	0.80	4.0% (-0.9–8.8)	0.11
**Job Change**[d]	10.6% (6.7–14.2)	<0.001	15.7% (10.9–20.5)	<0.001
**Married**	0.6% (-1.3–2.4)	0.53	-0.4% (-3.6–2.8)	0.81
**Family size** (per person)	-0.1% (-0.6–0.5)	0.86	-0.2% (-1.2–2.8)	0.72
**Family Income**				
≤138% FPL	6.5% (4.2–8.8)	<0.001	17.3% (13.4–21.2)	<0.001
139-249% FPL	5.1% (2.6–7.6)	<0.001	12.1% (8.5–15.7)	<0.001
250-400% FPL	2.6% (0.5–4.7)	0.02	8.3% (5.1–11.4)	<0.001
>400% FPL	ref.	-	ref.	-
**Year**				
2005-2009	ref.	-	ref.	-
2010-2013	-1.9% (-3.6– -0.1)	0.03	-1.2% (-4.2–1.7)	0.43
2014-2018	-0.9% (-2.9–1.1)	0.36	-4.2% (-7.4– -1.0)	0.01

FPL=federal poverty level; ref.=referent group
[a] Includes controls for all listed variables
[b] Insurance loss corresponds to change from any insurance coverage to uninsured at some point during the year
[c] Indicator for self-reported diagnosis of one or more of: coronary artery disease, myocardial infarction, stroke, diabetes, or emphysema
[d] Low education = no post-high school degree; job change = at least 1 job change, gain, or loss in year

© 2021 Albright BB et al. *JAMA Network Open.*

eTable 3. Weighted Linear Regression Models for Predictors of Catastrophic Health Expenditures (Family Expenses >10% Family Income) Among the Nonelderly (<65) Cancer Population, Medical Expenditures Panel Survey, 2005-2018

	**Expenses excluding premiums >10% income**		**Expenses including premiums >10% income**	
	**β (95%CI)**	***P* Value**	**β (95%CI)**	***P* Value**
**Age**				
<27 years	ref.	-	ref.	-
27-44 years	0.2% (-4.2–4.7)	0.93	-0.0% (6.3–6.2)	0.99
45-64 years	4.7% (0.3–9.1)	0.04	7.3% (1.6–12.9)	0.01
**Female Sex**	-0.3% (-2.4–1.7)	0.74	1.8% (-1.1–4.7)	0.23
**Race**				
White	ref.	-	ref.	-
Black	-6.2% (-8.8– -3.7)	<0.001	-10.9% (-14.2– -7.5)	<0.001
Other	-0.8% (-4.3–2.7)	0.66	-2.9% (-8.1–2.4)	0.28
**Hispanic Ethnicity**	-5.1% (-7.8– -2.3)	<0.001	-10.2% (-14.5– -5.9)	<0.001
**Major comorbidity**[b]	1.9% (-1.0–4.7)	0.19	3.1% (-0.5–6.8)	0.10
**Low Education**[c]	-6.8% (-10.8– -2.8)	0.001	-11.9% (-16.7– -7.1)	<0.001
**Job Change**[c]	-1.5% (-4.8–1.9)	0.39	-5.8% (-9.8– -1.8)	0.004
**Married**	3.2% (0.5–5.8)	0.02	9.0% (5.6–12.5)	<0.001
**Family size** (per person)	-1.4% (-2.2– -0.7)	<0.001	-2.6% (-3.5– -1.6)	<0.001
**Family Income**				
≤138% FPL	37.4% (33.2–41.6)	<0.001	44.3% (39.4–49.2)	<0.001
139-249% FPL	20.1% (17.0–24.8)	<0.001	35.1% (30.8–39.3)	<0.001
250-400% FPL	8.9% (6.7–11.2)	<0.001	24.5% (19.8–27.2)	<0.001
>400% FPL	ref.	-	ref.	-
**Year**				
2005-2009	ref.	-	ref.	-
2010-2013	-2.5% (-4.9– -0.1)	0.04	-0.04% (-3.9–3.2)	0.84
2014-2018	-3.0% (-5.3– -0.8)	0.008	0.4% (-2.8–4.5)	0.82

CHE = catastrophic health expenditures, considered at family-level as ratio of family out-of-pocket expenses alone or family out-of-pocket expenses plus premiums to family income; FPL=federal poverty level; ref.=referent group

[a] Includes controls for all listed variables

[b] Indicator for diagnosis of one or more of: coronary artery disease, myocardial infarction, stroke, diabetes, or emphysema

[c] Low education = no post-high school degree among those > 18 years old; job change = at least 1 job change, gain, or loss in year

© 2021 Albright BB et al. *JAMA Network Open.*

eFigure 1. CONSORT Diagram of Data Management and Study Population Selection for Study of Patients With Cancer Relative to Those Without Cancer, Medical Expenditure Panel Survey, 2005-18

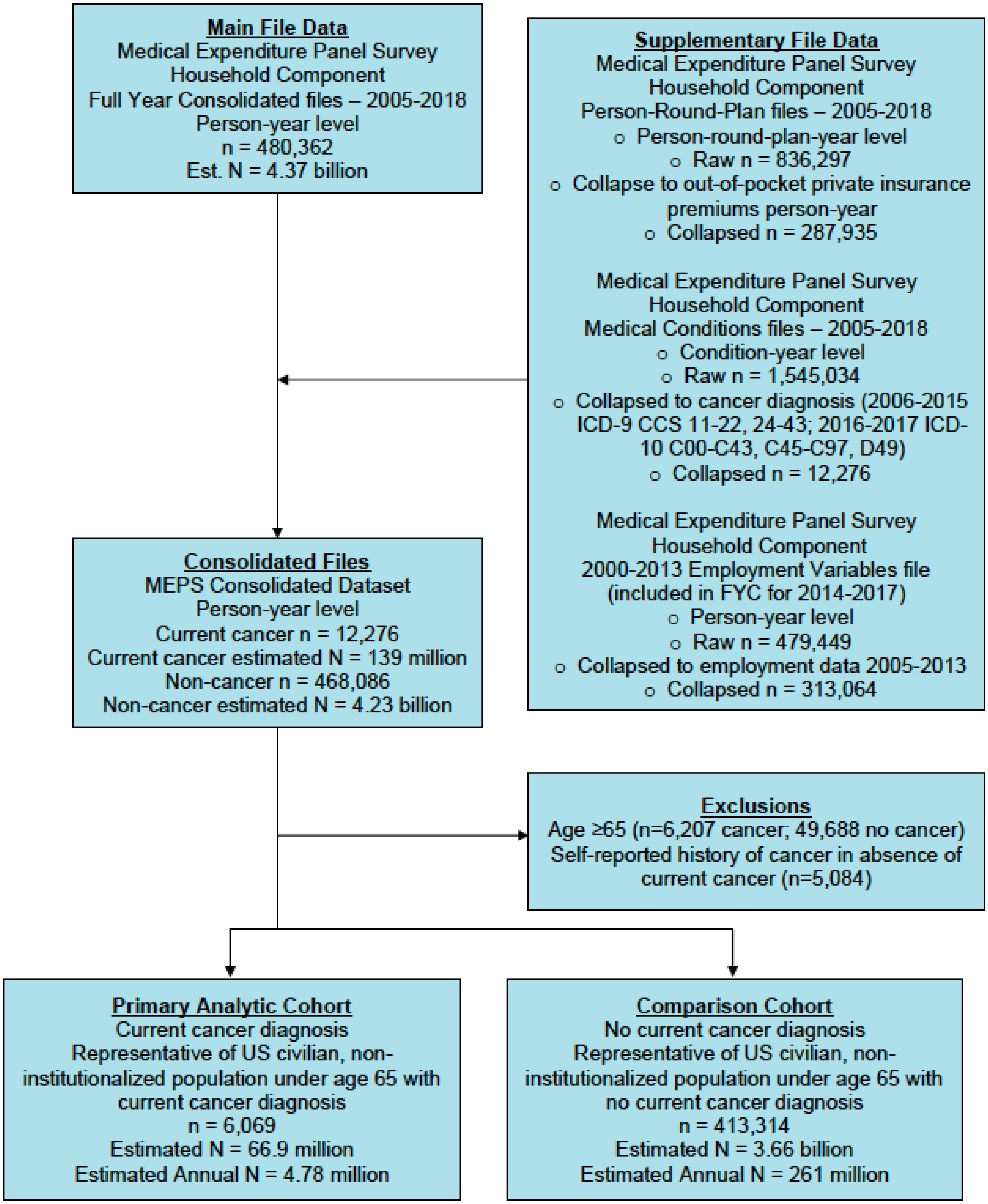

© 2021 Albright BB et al. *JAMA Network Open.*

eFigure 2. Trends in Insurance Churn Risks for the Nonelderly Noncancer Population, Relative to the Implementation of the Affordable Care Act, Medical Expenditure Panel Survey, 2005-2018

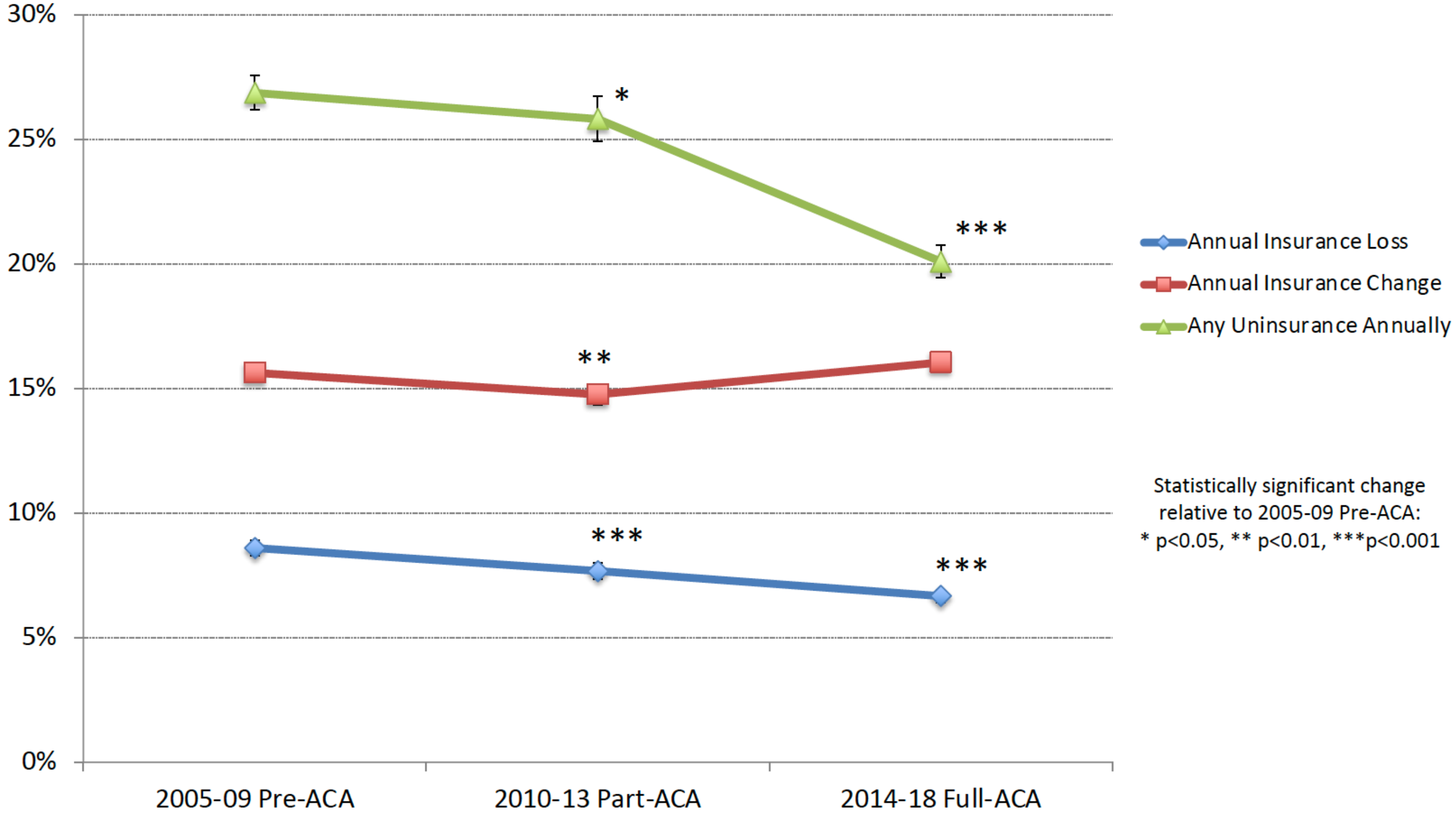

© 2021 Albright BB et al. *JAMA Network Open.*

eFigure 3. Trends in Catastrophic Health Expenditures (Ratio of Family Out-of-Pocket Expenses to Family Income) Risks for the Nonelderly Noncancer Population, Relative to the Implementation of the Affordable Care Act, Medical Expenditure Panel Survey, 2005-2018

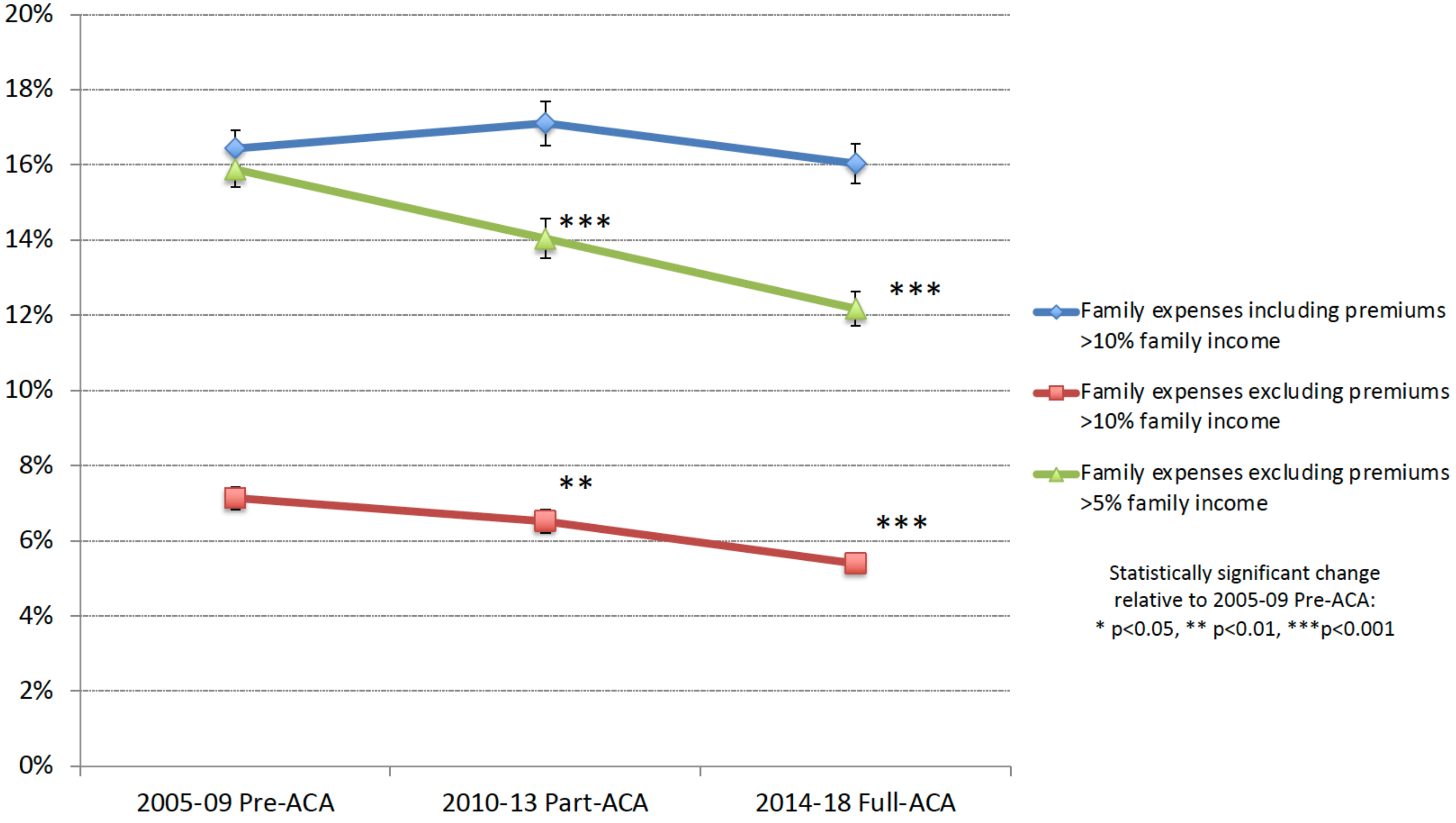

© 2021 Albright BB et al. *JAMA Network Open.*
